# Mortality in India established through verbal autopsies (MINErVA): Strengthening national mortality surveillance system in India

**DOI:** 10.7189/jogh.10.020431

**Published:** 2020-12

**Authors:** Anand Krishnan, Vivek Gupta, Baridalyne Nongkynrih, Rakesh Kumar, Ravneet Kaur, Sumit Malhotra, Harshal R Salve, Venkatesh Narayan, Ayon Gupta, on behalf of The MINErVA Network

**Affiliations:** 1 Centre for Community Medicine, All India Institute of Medical Sciences, New Delhi, India; 2 Dr Rajendra Prasad Centre of Ophthalmic Sciences, All India Institute of Medical Sciences, New Delhi, India

## Abstract

**Background:**

Following data access and storage concerns, Government of India transferred the management of its Sample Registration System (SRS) based mortality surveillance (formerly known as the Million Death Study) to an Indian agency. This paper introduces the new system, challenges it faced and its vision for future.

**Methods:**

The All India Institute of Medical Sciences (AIIMS), New Delhi, the new nodal agency, established the “Mortality in India Established through Verbal Autopsy” (MINErVA) platform with state level partners across India in November 2017. The network in its first three years has undertaken capacity building of supervisors conducting verbal autopsy under the SRS, established a panel of trained physician reviewers and developed three IT-based platforms for training, quality control and coding. Coding of VA forms started from January 2015 onwards, and the cause specific mortality fractions (CSMF) of the first 14 185 adult verbal autopsy (VA) records for 2015 were compared with earlier published data for 2010-2013 to check for continuity of system performance.

**Results:**

The network consists of 25 institutions and a panel of 676 trained physician reviewers. 916 supervisors have been trained in conducting verbal autopsies. More than 75 000 VA forms have been coded to date. The median time taken for finalizing cause of death on the coding platform is 37 days. The level of physician agreement (67%) and proportion of VA forms requiring adjudication (12%) are consistent with published literature. Preliminary CSMF estimates for 2015 were comparable with those for 2010-2013 and identified same top ten causes of death. In addition to the delay, two major challenges identified for coding were language proficiency of physician reviewers vis-à-vis language of narratives and quality of verbal autopsies. While an initial strategic decision was made to consolidate the system to ensure continuity, future vision of the network is to move towards technology-based solutions including electronic data capture of VAs and its analysis and improving the use of mortality data in decision making.

**Conclusion:**

MINErVA network is now fully functional and is moving towards achieving global standards. It provides valuable lessons for other developing countries to establish their own mortality surveillance systems.

Counting all deaths or a representative sample of all deaths with accurate determination of cause of death is the basic requirement of any national mortality surveillance system [1]. Information on mortality rates and causes is essential for choosing appropriate intervention measures and efficient resource allocation to improve population health [2,3]. Mortality data also helps monitor the achievement of targets related to mortality reduction for specific diseases [4]. Many disease control programs like non-communicable diseases, tuberculosis, malaria have identified mortality reduction goals and these are reflected in the Sustainable Development Goals (SDGs) [5]. The need for robust mortality surveillance systems for monitoring the progress towards attainment of SDGs is clear [6]. Thus, all countries must invest in setting up mortality surveillance systems including cause of death ascertainment.

Many deaths in developing countries occur at home and because of deficiencies in medical certification of cause of deaths, the use of Sample Vital Registration with Verbal Autopsy (SAVVY) has been recommended by WHO and found to be feasible [7]. In 2019, WHO reported that 11 health-related SDG indicators require cause-of-death data, yet less than one third of countries have high-quality data on cause of death [8]. Rao estimated that 65% of the estimated world population in 2019 reside in countries that lack data for reliably estimating population level cause-specific mortality [9].

India recognized the need for establishing a robust mortality surveillance system and has made considerable progress in its civil registration for births and deaths [10]. The paucity of valid and reliable data on causes of death, led to the piloting and addition of a verbal autopsy component to the routine demographic system under the Office of Registrar General of India (ORGI)’s Sample Registration System (SRS) in 1998. This was followed by a full roll out and the implementation was managed by the Centre for Global Health Research (CGHR) at the University of Toronto through a network of 300-400 physician reviewers and experts from various Indian academic partners. It aimed to cover 1 million deaths from 1998 to 2014 and details of this have been published earlier [11]. This effort resulted in availability of mortality estimates for major diseases in children and adults for India. Unquestionably, these estimates promoted evidence-based policy decisions, including a better appreciation of Non Communicable Diseases (NCDs) as a major contributor for deaths and disabilities in India [11,12]. However, the estimates generated by this Million Death Study (MDS) differed from the government or WHO estimates based on deaths recorded within the government systems for diseases like malaria [13-16].

A major criticism of the system under the MDS has been the delay in availability of information of cause of death in public domain which has hampered effective public health action. The delay is likely to be contributed by causes both due to the inherent delays at SRS system level, and due to coding under the MDS platforms [11]. Other criticisms include the management of the system by a foreign agency and it being seen as a research function rather than being an integrated part of national mortality surveillance system. These concerns, along with a need for strengthening national capacity to run such essential public health platforms, prompted the Government of India to select the All India Institute of Medical Sciences (AIIMS), New Delhi as its technical partner to integrate SRS-VA based cause of death ascertainment into routine practice and improve efficiency. A Memorandum of Understanding (MoU) was signed between the two institutions in March 2017 for coding deaths from January 2015 onwards. This led to the launch of Mortality in India Established through Verbal Autopsy” (MINErVA) Network in November 2017.

Establishing large scale national mortality systems in developing countries has its own challenges related to cost, capacity, culture and finally ensuring continuity and sustainability as reported from Indonesia and China [17,18]. Sharing such experiences and lessons are useful to other countries. Through this paper, we describe the MINErVA network, its platforms; the challenges and lessons faced in migrating to the new system and most importantly, our vision for the future.

## UNDERSTANDING THE CONTEXT

The erstwhile MDS and the MINErVA platform are both built on the existing publicly funded mortality surveillance framework of the SRS. We describe the SRS and the strategic decisions taken during the initial transition period to understand the context in which the MINErVA network took birth.

The SRS is a large, routine demographic survey and the primary system for the collection of Indian fertility and mortality data since 1971. The current sample, based on the 2011 Census frame, is operational in 8850 sample units (4961 rural and 3889 urban) covering about 7.9 million population, spread across all states and union territories. The field work consists of continuous enumeration of births and deaths in sample units by a resident part-time enumerators (PTE) and an independent survey every six months by SRS supervisors. The data obtained by these two independent functionaries are matched, re-verified in case of discrepancies and an unduplicated count of births and deaths is obtained. Verbal Autopsy (VA) is conducted by the SRS supervisors once the line list of deaths is finalized [19]. This process which often takes up to a year, is a primary cause of delay in the system [11]. Keeping in mind that the VAs are done as a part of other responsibilities by lay workers, the VA forms have been kept simple and are of two-pages in length with a narrative section to be filled in local language. These paper-based VAs are collected by respective state offices and mailed to the central office where they are scanned and handed over to the agency managing the VA coding process. Delays are common during this process.

The first thought of the AIIMS team on assumption of management of this system was to understand the current system so that it could be built upon. Our immediate challenge was a lack of access to the MDS physician network, training manuals and coding platforms, due to concerns related to Intellectual Property Rights expressed by both the parties concerned (CGHR and ORGI). This warranted the establishment of a new physician reviewer network and de novo development of web-based physician training and coding platforms, delaying the commencement of physician review of VA forms. However, it also provided us with an opportunity to rethink the tools, processes and platforms. Our initial rapid assessment indicated the need to revise the whole system, from the VA tools used, conduct of the VA to the coding process. However, bearing in mind our own capacity limitations, we took a considered and deliberate decision to replicate the existing system to preserve continuity and provide comparability with the erstwhile system before embarking on modifications to improve efficiency and international comparability. This decision was also driven by the fact that there had already been a considerable delay in the coding process due to the ongoing change in management and, revision of tools and processes might further delay the release of data into public domain. Some other key decisions pertaining to the network related to working through institutional mechanisms rather than with individuals and having state level network co-ordinators. It was decided that the platform would focus on mortality surveillance and research on newer methods of mortality surveillance.

## THE MINErVA NETWORK AND ITS PLATFORMS

**Figure 1:**
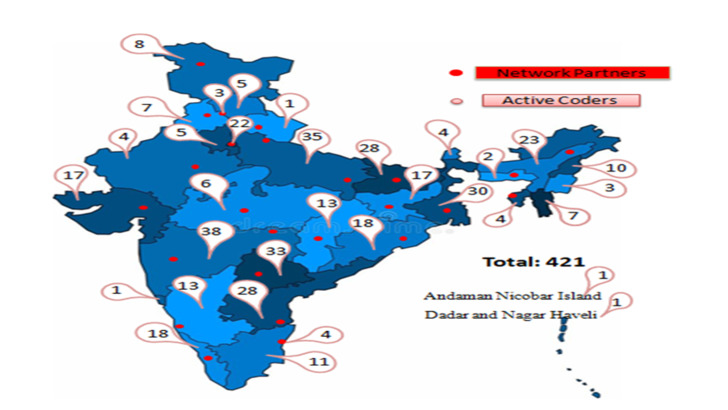
Geographical distribution of MINErVA network institutions and physician reviewers.

The MINErVA network has four components – AIIMS Technical Support Unit (ATSU), Network Partners and Physician reviewers and Technical Advisory Group (TAG), and three platforms – training, quality control and coding). The ATSU liaises with the ORGI and network partner medical colleges, manages administrative and financial issues, develops training programs, conducts training of trainers and, most importantly, deliberates on all technical matters internally before it is taken to partners and TAG. It also meets with the ORGI team to review the activities and their progress. The co-location of the ATSU team within the ORGI premises allows closer coordination and understanding of process flows. The composition of the team can be found at https://causeofdeathindia.com/about-us/atsu-team. The network currently comprises of 24 regional institutions with AIIMS-New Delhi as the 25^th^ institution (**Figure 1**). Details of MINErVA Network members are listed in Table S1 in the **Online Supplementary Document**. Network partner institutions carry out state level activities to enroll additional physician reviewers, train SRS supervisors and enumerators in doing verbal autopsies, make field visits for quality control and disseminate the findings of the network to state level stakeholders. The network meets annually, to review progress and prepare an annual plan and presents these to the TAG for its guidance. The TAG consists of eminent public health experts, clinicians, social scientists, and statisticians as well as nominees from WHO and Government of India stakeholder institutions. It reviews all major decisions taken by the network and provides external oversight. It approves the vision and mandate which the network prepares for itself.

A nationwide physician reviewer network with adequate linguistic diversity has been established to review VA forms and assign cause of death (CoD) based on ICD-10 codes. A total of 1676 physicians had registered in the system by March 2020, out of which 676 have completed the training in verbal autopsy. 652 physicians have registered as physician reviewers with 421 physician reviewers actively involved in coding the forms. One-third of current registered physician reviewers are postgraduates with a median experience of 7.5 years after medical graduation and 28% reported previous experience of coding deaths under the MDS. Language proficiency and language wise load of VA forms for physician reviewers is depicted in **Table 1**. The mismatch between numbers of the VA forms with narrative in a particular language and the language proficiency of the physician reviewers was high for Kannada (form to reviewer ratio of 117), Malayalam (96) and Odia (80) and least for English (13) and Gujarati (6). We also have problems of sufficiency of physician reviewers in the less spoken languages, especially of northeast India.

**Table 1:** Language wise distribution of verbal autopsy (VA) forms and physician reviewers under the MINErVA network

Language	% share of VA forms by language* (n = 36778)	Language proficiency of physician VA physician reviewers(N = 421)	Forms: physician reveiwer ratio
Hindi	47.2	280	62
English	15.2	419	13
Odia	5.2	24	80
Malayalam	5.2	20	96
Marathi	5.1	46	41
Telugu	4.9	55	33
Kannada	4.8	15	117
Bengali	4.6	66	26
Tamil	3.8	19	73
Assamese	1.8	30	23
Punjabi	0.7	9	27
Manipuri	0.6	4	57
Khasi	0.4	2	71
Gujarati	0.3	20	6
Others†	0.2	12	6

VA – verbal autopsy

*Provisional, subject to final reconciliation

†Urdu, Sikkimese, Kashmiri, Maithili, Nepali, Bodo, Dogri, Mizo

The three information technology (IT) platforms were developed de novo and their features are depicted in **Table 2**. The online training and advocacy platform is hosted at www.causeofdeathindia.com and is in public domain. This platform is used to train physicians in assigning cause of death based on International Classification of Diseases 10th revision (ICD-10) codes, from verbal autopsies. The freely available training package comprises of six audio-visual modules with an end-module evaluation. On successful completion, users get an auto-generated certificate and are provided an option to enroll as a physician VA reviewer in the MINErVA network. Those who exercise this option are required to upload their medical degree and sign a data non-disclosure agreement. In addition, the website hosts important updates on the activities of the MINErVA network, and links to important documents and publications on mortality surveillance. We developed a quality assurance platform to check the extracted data for transcription errors and rectification prior to uploading the forms for coding by physicians. As described earlier, paper-based VA forms are digitized using Intelligent Character Recognition (ICR) software by the Electronic Data Processing (EDP) division of the RGI. The scanned images and corresponding data files are then handed over to ATSU, which is physically located in the same office for running through the QA platform.

**Table 2:** Brief description of MINErVA IT Platforms

Component	Domain/Access	Purpose	Innovations /New features
Training & Advocacy Platform: www.causeofdeathindia.com/	• In public domain	• Portal for dissemination of network activities	• Training in VA coding seen as capacity building and therefore in public domain
	• Access available after login	• Hosts the training program for assigning CoD (ICD-10 codes) from VA records for physicians	
		• Registration of interested physician reviewers	• Medical students and non-physician health researchers can also access training modules
Quality Assurance Platform	• Only for RGI/ ATSU Staff	• Extraction and digitization of VA paper forms and quality check for errors	• Quality assurance done in partnership with ORGI
		• Language, field completion and gender data checks	
VA Coding Platform: http://minervacoding.aiims.edu.in/	• All registered physician reviewers of Minerva Network through a login password system	• Training on use of coding platform with actual SRS-VA records	• Grading of narratives
		• Allocation of VA records to physician reviewers/ adjudicators	• Use of a third reviewer rather than reconciliation in event of disagreement on CoD between 2 physician reviewers
		• Matching and finalization of ICD-10 codes	• Matching of physician assigned CoD on basis of WHO VA code categories
		• Technical and administrative support to physician reviewers	• Use of dashboards to improve transparency and monitoring by state supervisors

CoD – cause of death, SRS-VA – Sample Registration System

**Figure 2:**
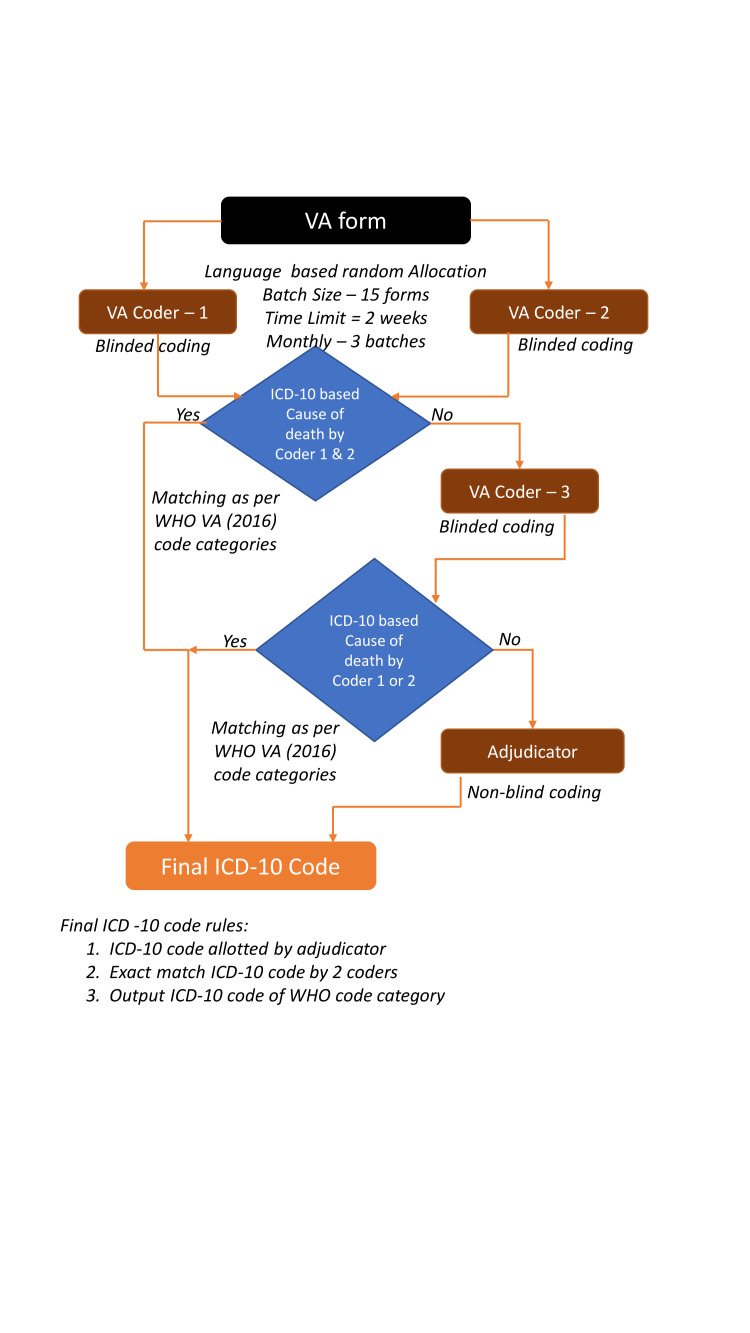
Verbal Autopsy coding process flow diagram.

The VA Coding Platform is hosted at http://minervacoding.aiims.edu.in/ and allows password controlled access to registered physician reviewers of the MINErVA network. VA forms are randomly allocated to individual physician reviewers by their language proficiency. Each VA form is independently reviewed by at least two physician reviewers who are blinded to each other’s diagnosis. The system matches agreement between them based on WHO VA (2016) code categories. In case of disagreement, the form is allocated to a third independent and blinded reviewer and if still not resolved, is allocated to an expert adjudicator for final assignment of cause of death. The VA coding process flow diagram is depicted in **Figure 2**. Physician reviewers are asked to grade the quality of the narrative portion of the VA form. The platform also has individual physician and state-level supervisor dashboards, detailing monthly coding workload and performance in terms of VA forms coded and individual physician reviewer agreement with the final assigned cause of death. We opted for an additional layer of third reviewer in case of disagreement between two physician reviewers instead of a reconciliation mechanism for operational simplicity.

## ACTIVITIES UNDERTAKEN UNDER MINErVA

**Capacity building:** The online physician reviewer training platform was launched in November 2017. In addition to physician coder training referred to above, nationwide training of SRS supervisors who conduct VA interviews was undertaken. The training consisted of a two-day program inclusive of a field component and was conducted in a phased manner by master trainers from network partners in close coordination with local ORGI staff. This training has to be undertaken in the short window between two rounds of surveys and so was done in two batches. A total of 916 SRS supervisors (>90% of total) underwent training. Only 10% of them reported attended any training in VA methods earlier. Our post-training assessment showed that the trainees improved from a baseline mean score of 6.1±2.8 to 8.6±1.2 (out of a total of 15). Only 28% of participants scored >60% on the pre-test while 62.5% scored >60% marks in the post test. The training module was rated as very useful by all the participants.

**Figure 3:**
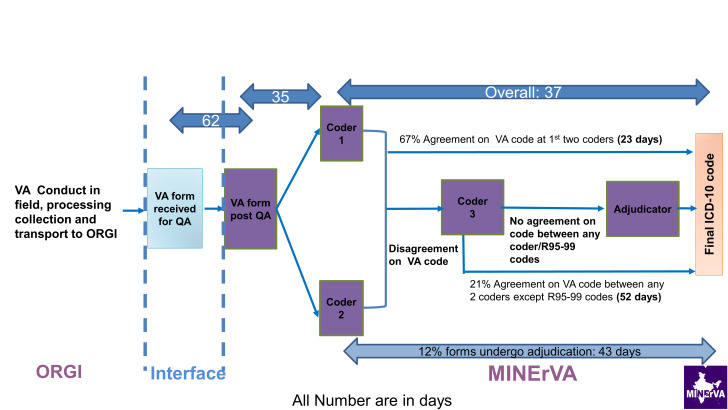
Timelines in physician review of cause of death under Sample Registration System – Virtual Autopsy (SRS-VA) system.

**VA coding:** The coding platform was launched in November 2018 and by March 2019, the platform had stabilized, and the coding process streamlined. At the time of writing (August 2020), about 75 000 deaths have been coded. On an average, 35 days is taken to allocate a VA form to an individual (**Figure 3**). This delay is largely due to the language proficiency mismatch alluded to above and a monthly ceiling of forms imposed on physician reviewers to ensure quality of coding. However, once allotted to a reviewer, it takes an average of 3-5 days for coding. The median interval between uploading a form on the coding platform to arrive at a final ICD code (including third coding or adjudication as necessary) was 37 days. On an average, two-thirds of the forms were finalized at the level of first two physician reviewers. Of the balance one third, 21% were finalized at third reviewer level and 12% forms were referred for adjudication. This is consistent with what has been reported under the MDS earlier as well as other VA systems [20,21].

## INTERIM ANALYSIS OF CAUSE OF DEATH

We compared our results of cause specific mortality fraction (CSMF) for first 14 185 adult deaths with a final ICD code in 2015 with the earlier published report for the year 2010-2013 [22] to establish that the shift to a new platform has not resulted in any major changes in cause of death patterns. To maintain consistency, we maintained the disease grouping uses in the 2010-13 report. The two CSMFs were quite comparable especially for injuries and non-communicable disease groups as seen in **Table 3**. There was a decrease (about 4%) in communicable disease proportions and an increase (about 5%) in unclassified deaths. The top ten cause of deaths in adults (15 years & above) remained the same with similar proportions.

**Table 3:** Comparison of cause specific mortality fraction (%) by major diseases and disease groups between 2015 and 2010-13 among adults 15 y & above

Major cause of death groups	2015 (partial) N = 14 185	2010-13 (full) N = 156 6791*
Communicable, maternal, perinatal & nutritional conditions	14.7	18.9
Injuries	10.6	11.2
Non-communicable diseases	55.5	55.7
Symptoms, signs & ill-defined conditions	19.2	14.2
**Top ten causes of death (%):**		
Cardiovascular diseases	27.2	27.1
Respiratory diseases	9.2	8.8
Malignant and other neoplasms	7.1	7.1
Digestive diseases	5.5	5.3
Unintentional injuries: Motor vehicle (MV) accidents	4.9	3.2
Fever of unknown origin	4.4	3.2
Tuberculosis	4.1	4.3
Unintentional injuries: Other than MV accidents	3.3	4.5
Diarrheal diseases	2.7	4.5
Intentional injuries: Suicide	2.2	3.0

*Source: Registrar General India, Centre for Global Health Research. Causes Of Death Statistics 2010-2013. Available: http://censusindia.gov.in/vital_statistics/VA_Report_2010-2013_Release.xlsx [22].

## FUTURE VISION AND STRATEGY

In its annual meeting in November 2019, the network partners and TAG members devoted considerable time to formulate a future vision and its strategy as they felt that the initial period of consolidation was over. The three broad thematic areas that were discussed were improving quality of VA and its coding, reducing delays, increasing the use of data. These are described below:

**Reduce delay in conduct of verbal autopsy and its coding:** Some steps suggested to the ORGI to reduce delay within its system include conduct of VAs by SRS supervisors at the time of enumeration of vital events without waiting for matching and moving from a paper-based system to an electronic data capture modality. The MINErVA network envisages a complete shift from paper-based forms to electronic data capture of all VA interviews using android app-based capture on mobile phones in 3-5 years’ timeframe. Our current coding capacity is enough to complete the annual load. However, the existing backlog of forms accumulated due to the transition needs a much higher system capacity, which we are currently working on. To circumvent the language proficiency issue, we plan to use translators to translate the narrative in such languages.

**Improving quality of verbal autopsy and its coding:** We are cognizant of currently higher proportion of unclassified deaths as compared to MDS but believe that this would be addressed over time as the quality of verbal autopsies available to physician reviewers improves due to the training of field staff which was undertaken by the network partners, and quality of coding by physicians also improves with experience. We plan to institute self-learning mechanisms as a part of the coding platform to encourage this process including online refresher training developed based on our analysis of common errors in cause of death coding. A webinar series including regular case discussion of difficult cases during such programs is also being instituted.

**Adopting global standards:** It is expected that as VA based systems are scaled up nationwide, a large number of VAs would enter the system. Transition to an automated Computer Coded Verbal Autopsy (CCVA) or a blended model of CCVA and PCVA methods with physicians signing off or modifying automated cause of death ascertainment would result in significant savings in time and resources [1]. We will therefore explore the use of technology including CCVA systems. The proposed shift within the SRS to complete electronic data collection also affords an opportunity to redesign the current VA tool. We are committed to research on this aspect, including the use of Machine Learning and Natural Language Processing tools to analyze narrative information, to better align the tool with WHO 2016 international standard VA and make it amenable to analysis by CCVA methods, while keeping local realities and needs in mind [23]. A move towards global solutions raises the need for a standardized approach and quality assurance for procedures of verbal autopsy and ascertainment of cause of death globally [24].

**Strengthen national capacity on VA:** Since inception, a conscious effort has been made to establish the MINErVA network in a manner which would permit institutional capacity building in the VA process at regional and state level through institutional partnerships. The network has a geographical presence in almost every state and UT of India and has ensured linguistic diversity. We plan to expand from medical college-based system to involve state health systems in our ambit. The aim is to create a sustainable and wide pool of trained manpower with adequate linguistic and geographic diversity to undertake VA activities in the country.

**Use of data for research and decision making:** A data sharing and security policy conforming with the standards outlined in India’s Personal Data Protection Bill 2019 will be formulated with representatives from Government of India stakeholders including ORGI. Storage and transmission of VA data through electronic means and access to this data will be governed by this policy. One of the limitations of mortality surveillance systems worldwide, is the poor link to action by the relevant stakeholders. Cause of death data in India is generated by the ORGI which is under the purview of the Ministry of Home Affairs while its primary users are Ministries of Health at central and state level. The MINErVA network is committed to establish strong and transparent linkages with all users and stakeholders of mortality data and has ensured their presence in its advisory group. Recognizing the limited access to this data for researchers, we plan to work with all concerned to develop an enabling data sharing policy to promote its use.

**Going beyond SRS:** The MINErVA Network aims to foster large-scale adoption of VA based methods for all deaths occurring outside hospitals and their integration with the Civil Registration and Vital Statistics Systems (CRVS). The small sample size of SRS is an impediment to the timely release of data as the results are released for three years for statistical stability. The SRS sample is also inadequately powered to make reliable estimates of child, maternal and adult mortality at state and regional levels [25,26]. The MINErVA network envisions to increase the footprint of VA based cause of death ascertainment systems beyond the SRS. Network partner institutes are encouraged to establish local level linkages and institute VA based mortality surveillance systems in local populations. At the national level discussions are being held with the Indian Council of Medical Research (ICMR) to scale up population coverage of VA based mortality surveillance systems to enable robust cause specific mortality estimates at state and regional levels.

## CONCLUSION

This paper describes the new platform developed using a collaborative approach to strengthen the cause of death ascertainment system through verbal autopsies in India. The network uses software packages developed in-house, using free open source applications for registering and training physician reviewers, quality assurance during data digitization and for coding of VA forms by physician reviewers. Despite initial challenges of developing the system, the system is performing well. It provides a replicable and scalable model for other developing countries where such systems are needed. The network will work with global experts to strengthen VA systems and is also ready to share its expertise with other countries who wish to move in this direction.

## Additional material

Online Supplementary Document

## References

[1] de Savigny D Riley I Chandramohan D Odhiambo F Nichols E Notzon S Integrating community-based verbal autopsy into civil registration and vital statistics (CRVS): system-level considerations. Glob Health Action. 2017;10:1272882. 10.1080/16549716.2017.1272882 28137194 PMC5328373

[2] Gouda HN Flaxman AD Brolan CE Joshi R Riley ID AbouZahr C New challenges for verbal autopsy: Considering the ethical and social implications of verbal autopsy methods in routine health information systems. Soc Sci Med. 2017;184:65-74. 10.1016/j.socscimed.2017.05.002 28501755

[3] World Health Organization. Improving the Quality and Use of Birth, Death and Cause-of-Death Information: Guidance for a Standards-Based Review of Country Practices. Geneva: WHO; 2010.

[4] Byass P Who needs cause-of-death data? PLoS Med. 2007;4:e333. 10.1371/journal.pmed.0040333 18031198 PMC2082647

[5] GBD 2015 SDG Collaborators Measuring the health-related Sustainable Development Goals in 188 countries: a baseline analysis from the Global Burden of Disease Study 2015. Lancet. 2016;388:1813-50. 10.1016/S0140-6736(16)31467-2 27665228 PMC5055583

[6] Alwan A Ali M Aly E Badr A Doctor H Mandil A Strengthening national health information systems: Challenges and response. East Mediterr Health J. 2017;22:840-50. 10.26719/2016.22.11.840 28177115

[7] Mudenda SS Kamocha S Mswia R Conkling M Sikanyiti P Potter D Feasibility of using a World Health Organization-standard methodology for Sample Vital Registration with Verbal Autopsy (SAVVY) to report leading causes of death in Zambia: results of a pilot in four provinces, 2010. Popul Health Metr. 2011;9:40. 10.1186/1478-7954-9-40 21819583 PMC3160933

[8] World Health Organization (WHO). World Health Statistics 2019: Monitoring Health for the SDGs, Sustainable Development Goals. Geneva; 2019. Available: https://www.who.int/gho/publications/world_health_statistics/2019/en/. Accessed: 3 February 2020.

[9] Rao C Elements of a strategic approach for strengthening national mortality statistics programmes. BMJ Glob Health. 2019;4:e001810. 10.1136/bmjgh-2019-001810 31681480 PMC6797430

[10] India RG. Vital Statistics of India Based on the Civil Registration System 2013. Available:http://www.censusindia.gov.in/2011-%0ADocuments/CRS_Report/CRS_Report2013.pdf.%0A. Published 2015. Accessed: 5 February 2020.

[11] Gomes M Begum R Sati P Dikshit R Gupta PC Kumar R Nationwide mortality studies to quantify causes of death: Relevant lessons from India’s million death study. Health Aff (Millwood). 2017;36:1887-95. 10.1377/hlthaff.2017.0635 29137507

[12] Krishnan A Das D Mortality surveillance in India: Past, present, and future. Indian J Public Health. 2019;63:163-4. 10.4103/ijph.IJPH_433_19 31552842

[13] Butler D Verbal autopsy methods questioned. Nature. 2010;467:1015. 10.1038/4671015a 20981062

[14] Kumar A Dua VK Rathod PK Malaria-attributed death rates in India. Lancet. 2011;377:991-5. 10.1016/S0140-6736(11)60379-6 21420546 PMC3883669

[15] Shah NK Dhariwal AC Sonal GS Gunasekar A Dye C Cibulskis R Malaria-attributed death rates in India. Lancet. 2011;377:991. 10.1016/S0140-6736(11)60378-4 21420545

[16] Valecha N Staedke S Filler S Mpimbaza A Greenwood B Chandramohan D Malaria-attributed death rates in India. Lancet. 2011;377:992-5. 10.1016/S0140-6736(11)60380-2 21420547

[17] Rao C Usman Y Kelly M Angkasawati T Kosen S Building Capacity for Mortality Statistics Programs: Perspectives from the Indonesian Experience. J Epidemiol Glob Health. 2019;9:98-102. 10.2991/jegh.k.190429.001 31241866 PMC7310751

[18] Liu S Wu X Lopez AD Wang L Cai Y Page A An integrated national mortality surveillance system for death registration and mortality surveillance, China. Bull World Health Organ. 2016;94:46-57. 10.2471/BLT.15.153148 26769996 PMC4709796

[19] Registrar General India. SRS Bulletin. New Delhi; 2019. Available: http://censusindia.gov.in/vital_statistics/SRS_Bulletins/SRS_Bulletin-Rate-2017-_May_2019.pdf. Accessed: 23 March 2020.

[20] Aleksandrowicz L Malhotra V Dikshit R Gupta PC Kumar R Sheth J Performance criteria for verbal autopsy-based systems to estimate national causes of death: Development and application to the Indian Million Death Study. BMC Med. 2014;12:21. 10.1186/1741-7015-12-21 24495287 PMC3912490

[21] Abegaz KH Berhe G Ferede S Physicians ’ agreement in determining the causes of death using verbal autopsy data in Eastern Tigray, Ethiopia. Res J Life Sci Bioinformatics, Pharm. Chem Sci (Camb). 2017;3:100-14.

[22] Registrar General India, Centre for Global Health Research. Causes Of Death Statistics 2010-2013. 2015. Available: http://www.cghr.org/wordpress/wp-content/uploads/COD-India-Report-2010-2013-Dec-19-2015.pdf. Accessed: 8 March 2020.

[23] Nichols EK Byass P Chandramohan D Clark SJ Flaxman AD Jakob R The WHO 2016 verbal autopsy instrument: An international standard suitable for automated analysis by InterVA, InSilicoVA, and Tariff 2.0. PLoS Med. 2018;15:e1002486. 10.1371/journal.pmed.1002486 29320495 PMC5761828

[24] Blau DM Caneer JP Philipsborn RP Madhi SA Bassat Q Varo R Overview and Development of the Child Health and Mortality Prevention Surveillance Determination of Cause of Death (DeCoDe) Process and DeCoDe Diagnosis Standards. Clin Infect Dis. 2019;69(Supplement_4):S333-S341. 10.1093/cid/ciz572 31598661 PMC6785670

[25] Gupta M Rao C Lakshmi PVM Prinja S Kumar R Estimating mortality using data from civil registration: a cross-sectional study in India. Bull World Health Organ. 2016;94:10-21. 10.2471/BLT.15.153585 26769992 PMC4709797

[26] Begg S Rao C Lopez AD Design options for sample-based mortality surveillance. Int J Epidemiol. 2005;34:1080-7. 10.1093/ije/dyi101 15911547

