# Supplementary material for: Mortality in India established through verbal autopsies (MINErVA): Strengthening national mortality surveillance system in India
Source: J Glob Health. 2020 Nov 8;10(2):020431. doi: 10.7189/jogh.10.020431 (PMC7688192; doi:10.7189/jogh.10.020431)
Supplement: Online Supplementary Document [file jogh-10-020431-s001.pdf]

<u>MINErVA Network details #</u>

1.	Subrata Baidya	Agartala Government Medical College	Agartala, Tripura, India
2.	Akhil Dhanesh Goel	All India Institute of Medical Sciences	Jodhpur, Rajasthan, India
3.	Pankaja R Raghav	All India Institute of Medical Sciences	Jodhpur, Rajasthan, India
4.	Arvind Singh Kushwaha	All India Institute of Medical Sciences	Nagpur, Maharashtra, India
5.	Pradeep Aggarwal	All India Institute of Medical Sciences	Rishikesh, Uttarakhand, India
6.	Surekha Kishore	All India Institute of Medical Sciences	Rishikesh, Uttarakhand, India
7.	Arun Kokane	All India Institute of Medical Sciences	Bhopal, Madhya Pradesh, India
8.	Arvind Kumar Singh	All India Institute of Medical Sciences	Bhubaneshwar, Odisha, India
9.	Binod Kumar Patro	All India Institute of Medical Sciences	Bhubaneshwar, Odisha, India
10.	Vikas Bhatia	All India Institute of Medical Sciences	Bhubaneshwar, Odisha, India
11.	Neeraj Agarwal	All India Institute of Medical Sciences	Patna, Bihar, India
12.	Shamshad Ahmad	All India Institute of Medical Sciences	Patna, Bihar, India
13.	Anjan Kumar Giri	All India Institute of Medical Sciences	Raipur, Chattisgarh, India
14.	Manisha Ruikar	All India Institute of Medical Sciences	Raipur, Chattisgarh, India
15.	Ajanta Deuri	Assam Medical College	Dibrugarh, Assam, India
16.	Nilesh Fichadiya	PDU Medical College	Rajkot, Gujarat, India
17.	R. G Mahajan	B J Medical College	Ahmedabad, Gujarat, India
18.	Sangeeta Kansal	Banaras Hindu University	Varanasi, Uttar Pradesh, India
19.	Dilip Kumar Das	Burdwan Medical College	Barddhaman, West Bengal, India
20.	Soumalya Ray	Burdwan Medical College	Barddhaman, West Bengal, India
21.	Malangori Abdulgani Parande	B J Government Medical College	Pune, Maharashtra, India
22.	Muralidhar Tambe	B J Government Medical College	Pune, Maharashtra, India
23.	Avneet Singh	Christian Medical College	Ludhiana, Punjab, India
24.	Shavinder Singh	Christian Medical College	Ludhiana, Punjab, India
25.	K J Kishore Kumar	Gandhi Medical College	Secunderabad, Telengana, India

26.	Vimala Thomas	Gandhi Medical College	Secunderabad, Telengana, India
27.	Mini S.S	Government Medical College	Thrissur, Kerala, India
28.	Sajna. M.V	Government Medical College,	Thrissur, Kerala, India
29.	Bhavna Sahni	Govt. Medical College	Jammu, Jammu & Kashmir, India
30.	K. C Premarajan	Jawaharlal Institute of Postgraduate Medical Education and Research	Puducherri, India
31.	S. Ganesh Kumar	Jawaharlal Institute of Postgraduate Medical Education and Research	Puducherri, India
32.	Animesh Jain	Kasturba Medical College	Mangaluru, Karnataka, India
33.	Vaman Kulkarni	Kasturba Medical College	Mangaluru, Karnataka, India
34.	G K Medhi	North Eastern Indira Gandhi Regional Institute of Health and Medical Sciences	Shillong, Meghalaya, India
35.	Himashree Bhattacharya	North Eastern Indira Gandhi Regional Institute of Health and Medical Sciences	Shillong, Meghalaya, India
36.	Star Pala	North Eastern Indira Gandhi Regional Institute of Health and Medical Sciences	Shillong, Meghalaya, India
37.	P. V. M. Lakshmi	Postgraduate Institute of Medical Education and Research	Chandigarh, India
38.	Tarundeep Singh	Postgraduate Institute of Medical Education and Research	Chandigarh, India
39.	Dewesh Kumar	Rajendra Institute of Medical Sciences	Ranchi, Jharkhand, India
40.	Vivek Kashyap	Rajendra Institute of Medical Sciences	Ranchi, Jharkhand, India
41.	K Ashok Kumar Reddy	Sri Venkateswara Medical College	Tirupati, Andhra Pradesh, India
42.	Dhiraj Kumar Srivastav	Uttar Pradesh University of Medical Sciences	Saifai, Uttar Pradesh, India
43.	Pankaj Kumar Jain	Uttar Pradesh University of Medical Sciences	Saifai, Uttar Pradesh, India

Institution and author names in alphabetical order.
